# Perceived Social Support and Anxiety among Chronic Hepatitis-C Patients

**DOI:** 10.12669/pjms.39.6.7412

**Published:** 2023

**Authors:** Muhammad Hafeez, Qurat Ul Ain Hafeez, Fuad Ahmad Siddiqi

**Affiliations:** 1 Muhammad Hafeez, FCPS (Medicine), FCPS (Gastro), FACG, FASGE (USA), FRCP (Edin), SCE Gastro (UK), CHPE (NUMS). Professor of Medicine, Bahria University Health Sciences Campus Karachi, Gastroenterologist & HOD Medicine, PNS Shifa, Karachi Pakistan; 2 Qurat Ul Ain Hafeez, BS Psychology Psychologist, PNS Shifa, Karachi Pakistan; 3 Fuad Ahmad Siddiqi, FCPS (Medicine), FRCP (Edin), FRCP (Glasgow), MACP (USA), Diploma in Medicine (AFPGMI), CHPE (NUMS) HOD Medicine, CMH Peshawar, Pakistan

**Keywords:** Perceived social support, Anxiety, Chronic Hepatitis C

## Abstract

**Objective:**

To study the relationship of perceived social support (PSS) and anxiety among patients of Chronic Hepatitis C (CHC).

**Methods:**

It is a cross sectional survey conducted from March 2021 to December 2021 in different hospitals of Islamabad and Rawalpindi. Sampling was done through purposive and snowball technique. Self-administered and standardized questionnaires were used. To analyze perceived social support and anxiety, perceived social support “scale” and Beck anxiety inventory were used in patients of Hepatitis C. Patients Polymerase Chain Reaction (PCR) positive HCV patients between the ages 31 to 50 years were included. Patients with comorbid conditions and other than 31 to 50 years of age were excluded from the study.

**Results:**

Out of 250, males were 185(74%) and females 65(26%). Ages were between 31 to 50 years. PSS in both males and females was 49.8 and 49 (p=0.63%) with anxiety level 44.63 and 56.18 (p=0.00) respectively. There was no significant gender differences on PSS but women had significantly higher on anxiety (*M* = 56.18, *SD* = 11.36) with moderate effect size (>.05). PSS had significant negative correlation with anxiety (*r* = -.31, *p* < .05).

**Conclusion:**

Anxiety is more common in females and perceived social support has negative correlation with anxiety in patients of Chronic Hepatitis C.

## INTRODUCTION

Chronic hepatitis C (CHC) is characterized as a systemic and a blood-borne illness.^1^ It has hepatic and extra-hepatic symptoms, along with high healthcare and social expenses making it a serious health concern worldwide.^2^ Across the world, 80 to 200 million people (representing around 2.4% of the total global population) are infected with hepatitis C virus each year.^3^,^4^ In 2015, 1.34 million fatalities from hepatitis were recorded worldwide. Over 40% of these people reside in Asia, China, Pakistan, India and Japan having significant population infected with CHC.^5^ Pakistan ranks second among the countries bearing the burden of chronic hepatitis with the national frequency of 4.8-6%,^6^-^8^ i.e. out of twenty Pakistanis one is infected by hepatitis C virus .^9^

The infectious nature of the hepatitis C, along with a tiresome and bustling hospital environment, may lead to social detachment and loneliness among the patient. It might be difficult for the people around them specially friends and families to provide comfort and support. To treat them like normal individuals, social support is necessary for such patients to have quick recovery and coping with the illness.^10^ CHC may impact adversely on the quality of life of patients. Worse socialization, isolation and relationship issues along with elevated levels of stress and anxiety are common among patients who are aware about the progression of their illness and also because of physical tiredness or feelings of incompetence.

A meta-analysis of CHC patients, who are even untreated, showed a significant level of anxiety. CHC is widely documented to cause symptoms such as sadness, irritability, anxiety, agitation, loss of appetite, tiredness, sleep disorder and impairment of cognition.^11^ The stress buffering theory of social support is utilized to conceptualize this research, implying that social support may alleviate the negative impacts of stress on health outcomes. In Pakistan very few research studies have been conducted on the relationship between PSS and anxiety among CHC patients. This research would contribute to a better understanding of the impact of PSS on the anxiety among the CHC patients.

## METHODS

The current study was based upon cross sectional survey research and used quantitative approach. It was conducted in joint cities of Pakistan i.e., Islamabad and Rawalpindi from May 21 to December 21 after the ethical committee of the main Hospital vide letter no A/28/86/EC/45/2022. Purposive and snow ball sampling technique was used. Self-report measures were used for data collection. Demographic sheet explain all demographic variables i.e. age, gender, marital status, qualification and family system. A pilot study was carried out to examine the cronbach alpha reliabilities of the scales used both for PSS scale and Beck anxiety inventory.

PSS scale is a self-report measure and consists of 12 items. Responses were made on seven point scale. The sum of the responses determines the levels of PSS. This scale consists of three sub scales i.e. family, friends and significant others support. The Cronbach alpha is 92 which indicates strong internal consistency.^12^ Beck anxiety inventory is self-reported scale with 21 items. Responses were recorded on four point scale ranging from zero to three. Total score was calculated by adding the sum of all the responses, which ranges between zero and 63. Higher scores (above 36) are the indicator of severe anxiety, 0-21 indicates low anxiety and 22-35 indicates the moderate levels of anxiety among individuals. This scale has alpha coefficient of 0.89 that represents the strong internal consistency.^13^

Before the administration of questionnaires participants were informed about the purpose of present study. Written consent was obtained from the participants and they were assured that the information received from them will be only used for research purpose and will remain confidential. Only willing participants were included in the study. Participants were asked to read the instructions of each scale carefully and provide their honest response while rating the statements. After the completion, data was analyzed by using SPSS (Statistical Package for Social Sciences (IBM-SPSS, Version 21).

## RESULTS

The present study was based to see the impact of perceived social support and anxiety among CHC patients. For this purpose a sample of 250 participants was collected from different hospitals located in Rawalpindi and Islamabad. Psychometric properties were established and found that all data was normally distributed and satisfy the assumption of normal probability (i.e., Table-I). Alpha reliability analysis was conducted and revealed satisfactory levels of both scales (i.e., Table-I). Cronbach (1950) indicated 70 as an acceptable reliability coefficient in defining consistency of instruments. Demographic details are shown in Table-I.

**Table-I:** Socio-Demographic Characteristic of Variables (N=250).

Variables	N	%
***Gender***		
Men	185	74
Women	65	26
***Age***		
31-40	53	21
41-50	197	79
***Marital Status***		
Un-Married	9	4
Married	241	96
***Family System***		
Joint Family System	163	65
Nuclear Family System	87	35

Out of 250 patients who participated in the study only 9(4%) patients were un-married. Descriptive analysis revealed that 65% of participant patients’ lives in joint family system, (Table-II).

**Table-II:** Correlation of perceived social support and anxiety (N=250).

Paired Samples Correlations			

	N	Correlation	Sig.
Perceived Social Support & Anxiety	250	-.310	.000

It represents the correlation coefficient between study variables. Perceived social support has significant negative correlation with anxiety in patients of chronic hepatitis c (*r* = -.31, *p* ≤ .05).

Illustrates differences on the basis of patient’s age and effect sizes in perceived social support and anxiety. Results show that patients reported non-significant differences in perceived social support and anxiety, (Table-III).

**Table-III:** Mean Difference in the Study Variables on the Basis of Patient’s Age (N=250).

Mean Difference in the Study Variables on the Basis of Patient’s Age (N=250)	31-40 (n=53)		41-50 (n=197)		Cohen’s d		

Variables	M	SD	M	SD	t(248)	p	
Perceived Social Support	49.32	12.65	49.82	12.47	.25	.79	.03
Anxiety	47.36	18.14	47.71	19.64	.11	.90	.01

***Note:*** M = Mean, SD = Standard Deviation, p ≤ .05.

While considering perceived social support, in joint family mean value was 51.3 however, in nuclear family it was 46.62 with significant P-value of ≤ 0.05. Anxiety was addressed in joint family mean value was 46.6 and nuclear family mean was 50.0 with insignificant P-value of 0.06, (Table-IV).

**Table-IV:** Mean Difference in the Study Variables on the Basis of Family System (N=250).

Variables	Joint Family (n=163)		Nuclear Family (n=87)		N	

	M	SD	M	SD	P	Cohen’s d
Perceived Social Support	51.3	13.94	46.62	8.39	.00	.41
Anxiety	46.6	20.81	50.0	15.74	.06	.25

***Note:*** M = Mean, SD = Standard Deviation, *p* ≤ .05.

## DISCUSSION

In the study PSS was found significantly negatively correlated with anxiety among CHC patients. Current findings are in the line with prior research that higher level of perceived social may be helpful to reduce anxiety among CHC patients.^14^ CHC patients may demand that their psychological needs to be met.^15^ Social connections are a vital source of social support. It aids in the self-management of chronic illnesses and plays a significant part in changing one’s lifestyle throughout sickness.^16^

Social interactions can give valuable social support by assisting with disease management efforts. In such circumstances, it’s critical to give extra attention to CHC patients’ emotional and social requirements.^15^ Gender differences in current study revealed that women experience more anxiety as compared to men hepatitis patients .However results were non-significant on level of perceived social support among participants. Previous studies are also in line with current findings. In terms of gender disparities, women with HCV appear to face more stigma, privacy concerns, treatment side effects, and a lack of involvement with health care than males.^17^

Gender appears to have an impact on depression, anxiety, and cognitive functioning, with women demonstrating more impairment than males. Substance abuse appears to have an impact on this outcome.^18^ In the current findings of the study age differences were non-significant. Differences were assessed on the basis of two levels of age groups including 31 to 40 years and 41 to 50 years of age. However results were found non-significant both on perceived social support and anxiety among chronic hepatitis C patients. There is also limited work available on age differences and psychological aspects of mental health chronic hepatitis C patients.^18^ Non-significant differences may be due to CHC a negative impact on their quality of life.^19^

A study of the psychological effects of hepatitis discovered that it is more stressful than losing your job or being divorced. The findings of the research revealed that hepatic patients had access to social assistance, but this help was less than what they need. Marital status also plays an important role with respect to enhancing social support and reducing anxiety. Findings of the study revealed that married persons reported higher level of PSS, whereas unmarried reported higher level of anxiety among chronic hepatitis C patients.

Breakups or severe stresses on sexual interactions, which were experienced by around 17% of the participants and the loss of ties with family members, which were experienced by another 16% of the participants, were the most painful outcomes. Our findings are similar with previous research concentrating on HIV/AIDS or TB patients who also felt rejected by family and friends.^20^ Social support is frequently cited as an important component of healthy relationships and mental well-being. Having a network of relatives and friends to whom you may turn in times of need is what social support is all about.^21^

The significance of having a robust social support network is frequently discussed by psychologists and other mental health experts. Experts typically advise people to depend on their friends and family for assistance when attempting to achieve their objectives or deal with a crisis. Findings of the study also showed the differences on the basis of family system in PSS and anxiety among chronic hepatitis C patients. Results revealed that CHC patients who belonged to extended family system reared higher level of PSS, whereas patients who belonged to nuclear family system reported more level of anxiety. Family members receive emotional, social, and financial support from their families.^21^-^22^

A high-functioning family assists its members in maintaining communication, emotional, and behavioral control, as well as problem-solving and coping strategies. A disease like CHC is serious and debilitating, and it takes a toll on the caregivers’ emotional and financial well-being. As patient age, their requirements for caregiving rise, and social ties in other domains, such as the job, become less prominent in their life; family bonds may become even more crucial to their well- being.^21^,^22^

Chronic hepatitis C leads to depression in most of the patients and its treatment helps in improvement of quality of life.^23^,^24^ In the study the new role of perceived social support has been stressed upon that may help the patients of chronic illness like hepatitis C better in coming out of the crises in addition to pharmacological treatment by reducing the anxiety.

### Limitation:

Limited age group is included in the study. Different stages of the chronic hepatitis C and its correlation with anxiety and perceived social support may have different findings.

## CONCLUSION

Anxiety is common among the female patients of CHC despite an equal PSS. Anxiety is negatively correlated with PSS. Perceived social support may help in better coping up the chronic conditions like Hepatitis C by allaying the anxiety. However further studies required to strengthen the concept.

### Author’s Contributions:

**MH:** Conceived idea, data collection, preparing the manuscript and final editing.

**QA:** data collection, preparing the manuscript.

**FAS:** Helped in data collection, article writing, review and final editing.
